# Supplementary material for: Congenital Malformations in a Holstein-Fresian Calf with a Unique Mosaic Karyotype: A Case Report
Source: Animals (Basel). 2020 Sep 10;10(9):1615. doi: 10.3390/ani10091615 (PMC7552221; doi:10.3390/ani10091615)
Supplement: Supplementary file 1 [file animals-10-01615-s001.pdf]

Supplementary material

Table S1. Analysis of chromosome or chromatid breaks and presence of micronuclei in *in vitro* cultured fibroblasts originated from the affected calf and a heathy control calf.

Animal	Metaphase spreads		Interphase nuclei	
	**Total number**	**Number of the spreads with chromatid/chromosome break or gap**	**Nuclei without micronucleus**	**Nuclei with micronucleus**
**Affected calf**	100	0	998	2
**Control calf**	100	0	999	1
